# Contextual and individual assessment of dental pain period prevalence in adolescents: a multilevel approach

**DOI:** 10.1186/1472-6831-10-20

**Published:** 2010-08-13

**Authors:** Marco A Peres, Karen G Peres, Antônio C Frias, José Leopoldo F Antunes

**Affiliations:** 1 Oral Epidemiology and Public Health Dentistry, Post-graduate Program in Public Health, Department of Public Health, Universidade Federal de University of Santa Catarina, Florianópolis, Brazil; 2 Department of Social Dentistry, Faculty of Dentistry, Universidade de São Paulo, São Paulo, Brazil; 3 Escola de Artes, Ciências e Humanidades, Universidade de São Paulo, São Paulo, Brazil

**Keywords:** dental pain, epidemiology, oral health, socioeconomic factors, multilevel analysis

## Abstract

**Background:**

Despite evidence that health and disease occur in social contexts, the vast majority of studies addressing dental pain exclusively assessed information gathered at individual level.

**Objectives:**

To assess the association between dental pain and contextual and individual characteristics in Brazilian adolescents. In addition, we aimed to test whether contextual Human Development Index is independently associated with dental pain after adjusting for individual level variables of socio-demographics and dental characteristics.

**Methods:**

The study used data from an oral health survey carried out in São Paulo, Brazil, which included dental pain, dental exams, individual socioeconomic and demographic conditions, and Human Development Index at area level of 4,249 12-year-old and 1,566 15-year-old schoolchildren. The Poisson multilevel analysis was performed.

**Results:**

Dental pain was found among 25.6% (95%CI = 24.5-26.7) of the adolescents and was 33% less prevalent among those living in more developed areas of the city than among those living in less developed areas. Girls, blacks, those whose parents earn low income and have low schooling, those studying at public schools, and those with dental treatment needs presented higher dental-pain prevalence than their counterparts. Area HDI remained associated with dental pain after adjusting for individual level variables of socio demographic and dental characteristics.

**Conclusions:**

Girls, students whose parents have low schooling, those with low *per capita *income, those classified as having black skin color and those with dental treatment needs had higher dental pain prevalence than their counterparts. Students from areas with low Human Development Index had higher prevalence of dental pain than those from the more developed areas regardless of individual characteristics.

## Background

Dental pain is described as pain originating from innervated tissues of the tooth or immediately adjacent to it [1]. It is a subjective oral health indicator caused mainly by dental caries and should become uncommon when oral health improves [2]. Conditions such as erosion, trauma, and exfoliation of primary teeth can also cause dental pain [3]. In low-to-middle income countries, most caries remain untreated, and dental care may not be easily available and is not universally free in most of these countries [4]. Most international data on dental pain have reported period prevalence more than point prevalence, and range between around 10 and 30% depending on the case definition and assessment methods adopted [5]. Period prevalence refers to the number of persons known to have had pain at any time during a specified period, usually 6 months in dental pain studies, while point prevalence refers to the number of persons with pain at a specified point in time [6].

When children and adolescents are taken into account, dental pain may be of social concern because it may cause suffering, sleep disturbances, diminish social activities, and increase school absenteeism. Therefore, dental pain potentially reduces the quality of life [7-9]. Reducing the population level of dental pain and the number of days absent from school, employment, and work owing to pain of oral and craniofacial origin are targets of Global Goals for Oral Health 2020 [10], and, consequently, societal efforts must be applied to achieve these goals.

The epidemiological literature about dental pain is scarce and has often been studied by adopting a point of view limited to biological aspects [11,12]. Few studies addressing social and demographic determinants of dental pain have reported strong association with poor family socioeconomic status [12-14] and cumulative episodes of poverty in the life course [12]. However, the relationship between dental pain and gender is uncertain and the link between dental pain and race/ethnicity has barely been investigated [12].

Despite evidence that health and disease occur in social contexts, the vast majority of studies addressing dental pain exclusively assessed information gathered at individual level. Multilevel studies in the dental public health dentistry literature are rare and have focused on dental caries [15], tooth loss [16-18], unsound teeth and periodontal pockets [19], and dental injuries [20]. We are unaware of any epidemiological study on dental pain adopting a multilevel approach. This is of concern, and the epidemiologic literature acknowledges the fallacy of studies that draw inferences at the contextual level by exclusively assessing individual-level data [21].

By considering that multilevel analysis is a suitable approach to take social contexts into account as well as individual-level information, the aim of this study was to assess the association between dental pain and Human Development Index (HDI, contextual) as well as individual socio demographic and dental characteristics in Brazilian adolescents. In addition, we aimed to test whether contextual Human Development Index is independently associated with dental pain after adjusting for individual level variables of socio-demographics and dental characteristics.

## Methods

This study was carried out in São Paulo, a metropolis with nearly 11 million inhabitants, the second largest city in Latin America, and the Capital of the most populous and industrialized Brazilian state. During the last decades, São Paulo experienced a relevant improvement in life expectancy and health indicators [22]. A major reform of the national health system in 1988 has boosted initiatives in dental public health and provision of dental care [23].

From September to November, 2008, the local health authority performed an oral health survey following international diagnostic criteria standardized by the World Health Organization [24]. All students aged 12 (179,674) and 15 years (184,537) in the city were eligible to participate in the study.

A total of 4,249 12-year-old and 1,565 15-year-old schoolchildren were examined, and their parents or guardians answered a questionnaire on socioeconomic and demographic conditions. The selection of participants followed a multistage, probabilistic sampling design aimed at allowing statistical inference on the outcomes of oral health with regard to the city as a whole and to each one of its 25 areas, which were geographically divided in 2005 by the local health authority for administrative purposes. These areas were the strata for the multistage selection of sample units, and schools were the primary sampling survey units for the random selection of schoolchildren. Each participating child was assigned a sampling weight corresponding to the inverse of its probability of selection.

As the oral health survey investigated several dental outcomes (dental caries, periodontal conditions, fluorosis, and malocclusion), sample size was calculated to exceed the minimum required for each outcome, based on the prevalence levels reported by a previous municipal oral health survey. Sample-size calculation considered a sample error ranging from 0.05 (prevalence of fluorosis) to 0.20 (dental caries index), a type I error of 5%, and a design effect of 1.25 for 12-year-old students and 1.50 for 15-year-old adolescents.

Refusals to participate were compensated by adding an addition of 30% participants, thus totaling to 4,249 12-year-old adolescents. Furthermore, aiming to allow stratified analysis and to increase statistical power, the original sample was enlarged by adding 1,565 15-year-old adolescents. The refusals were not replaced.

Dental examinations were carried out at the schoolyard, using natural light, periodontal probes (CPI probes), and plane mouth mirrors. Seventy-four specifically trained dentists performed the dental examinations; *kappa *statistics assessing inter examiner reliability previous to the fieldwork ranged from 0.70 (95% CI = 0.57 - 0.82) for dental fluorosis to 0.95 (95% CI = 0.94 - 0.96) for dental caries, which is satisfactory for this type of assessment [25].

### Outcome

Dental-pain period prevalence - the main outcome variable of this study - was assessed by the direct answer to the question "have you had toothache during the last six months?" Dental pain was originally recorded according to three categories - no, mild dental pain, and severe dental pain. We created a new binary variable by grouping mild and severe dental pain into one category.

### Explanatory variables

Explanatory variables assessed individual and contextual covariates. At the area level, the HDI presented the socioeconomic status. This index is a composite measurement encompassing information on income, education, and longevity, and calculated by governmental agencies [15] based on the most recent source of information on population, observing criteria established by the United Nations Development Program [15,26]. For analytical purposes, the HDI was categorically assessed, considering the median as the cutoff point.

At the individual level, demographic status was stratified by sex, age, and five categories of skin color/race group: Amerindians, Asian descendants, light- and dark-skinned blacks, and whites [27].

Socioeconomic position was assessed by the *per capita *family income, educational level of the parents, and type of school. Family income was divided into tertiles according to their frequency distribution in *Reais *(Brazilian currency), with cutoffs at half and a quarter of the Brazilian Minimum wage (BMW) *per capita*. The minimum wage is a standard for measuring income in Brazil, which broadly corresponded to 200 US dollars during the period of data collection. The classification of educational level of parents had cutoffs at 8 and 11 years of formal schooling, which in Brazil, corresponds to completion of primary and high school. As public schools do not collect tuition fee, the enrolment of children in private schools was used as a surrogate of improved socioeconomic status in epidemiologic studies on child health. Finally, the evaluation of dental status used the prevalence of untreated caries (having at least one tooth with untreated caries) and endodontic treatment need (having at least one tooth with indication for endodontic treatment) as covariates of dental pain.

#### Data analysis

Statistical analyses used Stata 10.0 (2007, Stata Corporation; College Station, Texas, USA). Data analysis considered the organization of the sample into strata and primary survey units as well as individual sampling weights estimated in the draft of complex survey data.

Maps of the city of São Paulo assessed the overlap of areas ranking higher dental-pain prevalence and poorer human development. The assessment of covariates for dental-pain prevalence used Poisson regression analysis; the prevalence ratio (PR) with 95% confidence intervals and *p *values were the outputs of the analysis.

**Figure 1:**
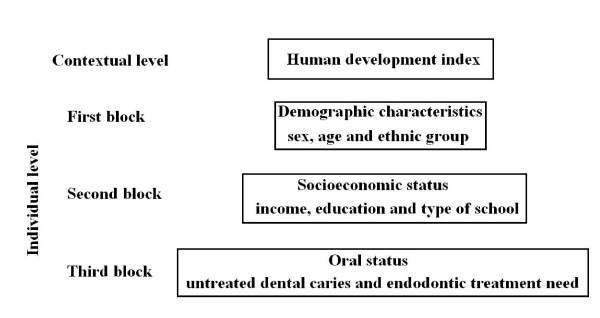
**Theoretical model of the relationship between contextual and individual characteristics on adolescents' dental pain**.

Poisson multilevel regression analysis used the scheme of fixed effects/random intercept [28], considering two levels of data organization: the examined schoolchildren and areas of the city. The hierarchical, multilevel analysis observed a conceptual framework to appraise covariates of dental pain, according to the model described by Victora *et al*. [29]. The HDI of residential areas was considered as the most distal determinant of dental pain. At the individual level, demographic characteristics were selected as the first block, thus allowing the assessment of all remaining covariates to be adjusted for the distribution of participants by sex, age, and ethnic group. Income, education, and type of school comprised the second block, thus allowing proximal covariates on the third block (dental status) to be adjusted for the differences in the socioeconomic status in the sample (Figure 1). All associations were adjusted for covariates positioned in the same and in the upper levels of the hierarchical model. Prevalence ratio for the Human Development Index was also estimated after controlled for all individual-level variables. Interaction between HDI and *per capita *family income was also assessed.

### Ethical issues

The study followed the national and international standards of ethics in research involving human participants; the study protocol was approved by the Research Ethics Committee of the sponsoring institution - Sao Paulo Health Authority (protocol No. 048/08 - March 18^th ^2008) - and written informed consent was obtained from parents and guardians of the participating adolescents.

## Results

**Figure 2:**
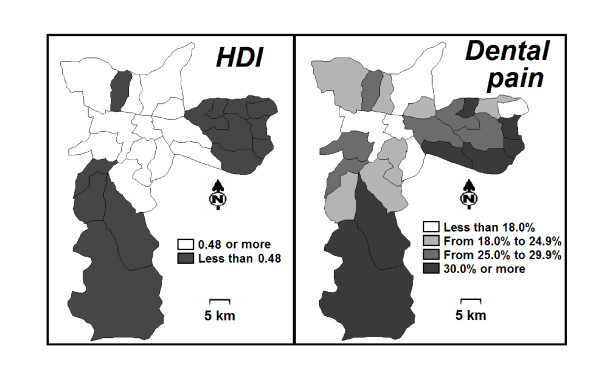
**Dental pain period prevalence among adolescents and human development index in areas of the city of São Paulo, Brazil**.

The response rate was 93.4 and 87.9% for 12- and 15-year-old schoolchildren, respectively. The main reason for refuses was the lack of written consents and school absenteeism when the study was carried out. Figure 2 shows the geographic distribution of the HDI and dental-pain prevalence among 12- and 15-year-old adolescents across the city. Higher levels of dental-pain prevalence were found to be concentrated in areas with lower values of HDI.

Table 1 describes the main characteristics of the sample; 3123 adolescents were studying in thirteen districts with low HDI (< 0.48) and 2692 adolescents from twelve districts with high IDH (≥0.48) composed the final sample. The proportion of boys and girls was similar and 3/4 of surveyed adolescents was 12 year-old; 1/3 of the sample was classified as black skin color, nearly 1/4 living in very poor family, and approximately 40% of the parent's participants had less than 8 years of study. Less than 10% of the adolescents studied in private schools and presented endodontic treatment need whereas nearly 40% of them had at least one tooth with untreated dental caries.

**Table 1:** Contextual and individual characteristics of studied adolescents

City level	n	%
*Human development index*		
< 0.48 (*n *= 13 districts)	3123	53.7
≥ 0.48 (*n *= 12 districts) All (*n *= 25 districts)	2692 5815	46.3 100.0

**Individual level**	**n**	**%**

**Demographic characteristics**		
*Sex*		
Boys	2792	48.0
Girls	3023	52.0
*Age (years)*		
12	4249	73.1
15	1566	26.9
*Skin color/race*		
Whites	2012	34.6
Dark-skinned blacks	668	11.5
Light-skinned blacks	3027	52.1
Asian descendants	72	1.2
Amerindians	36	0.6

**Socioeconomic status**		

*Per capita family income*		
< 1/4 BMW*	1262	21.8
1/4 |-- 1/2 BMW*	1595	27.4
≥ 1/2 BMW*	1491	25.6
Not informed	1467	25.2
*Father Educational level (years of study)*		
< 8	2397	41.3
8 |-- 11	968	16.6
> 11	1531	26.3
Not informed	919	15.8
*Mother Educational level (years of study)*		
< 8	2229	38.4
8 |-- 11	1067	18.3
> 11	1778	30.6
Not informed	741	12.7
*Type of school*		
Public	5260	90.5
Private	555	9.5

**Dental status**		

*Untreated dental caries*		
None	3583	61.6
≥ 1 teeth	2232	38.4
*Endodontic treatment need*		
None	5434	93.4
≥1 teeth	381	6.6

*BMW = Brazilian Minimum Wage

São Paulo, Brazil, 2008.

Dental pain affected nearly a quarter of the surveyed adolescents; dental pain was 33% less prevalent among adolescents living in more developed areas of the city than among those studying in less developed areas. Dental-pain prevalence varied according to individual demographic, socioeconomic, and dental status variables. Girls, dark and light-skinned blacks, those whose parents earn low incomes and with low schooling, and those studying at public schools presented higher dental-pain prevalence than their counterparts. In addition, untreated dental caries and endodontic treatment needs were strongly associated with dental pain. The highest prevalence of dental pain was found among adolescents with dental treatment needs. With regard to demographic and socioeconomic variables, adolescents with *per capita *family income lower than 1/4 BMW by month showed the highest dental-pain prevalence (32.8%), whereas adolescents studying at private school were on the opposite side of the scale (8.7%) (Table 2).

**Table 2:** Dental pain period prevalence* among adolescents according to the city and individual levels characteristics

City level	With	Pain
*Human development index*	N	%
*<*0.48	937	30.0
*≥ *0.48	544	20.2
All	1449	25.6

**Individual level**	**With**	**Pain**

	n	%

**Demographic characteristics**		
*Sex*		
Boys	539	19.3
Girls	946	31.3
*Age (years)*		
12	1016	22.6
15	469	24.4
*Skin color/race*		
Whites	403	16.6
Dark-skinned blacks	180	25.2
Light-skinned blacks	881	28.4
Asian descendants	11	13.7
Amerindians	10	23.7

**Socioeconomic status**		

*Per capita family income*		
< 1/4 BMW	418	32.8
1/4 |-- 1/2 BMW	461	29.0
≥ 1/2 BMW	241	14.8
Not informed	365	21.3
*Father educational level* *(years of study)*		
< 8	740	30.5
8 |-- 11	250	23.8
> 11	268	14.0
Not informed	227	23.6
*Mother Educational level* *(years of study)*		
< 8	695	31.6
8 |-- 11	274	23.6
> 11	336	15.2
Not informed	180	23.2
*Type of school*		
Public	1435	26.5
Private	50	8.7

**Dental Status**		

*Untreated dental caries*		
None	588	14.4
≥ 1 teeth	897	39.3
*Endodontic treatment need*		20.4
None	1222	68.8
≥ 1 teeth	263	

São Paulo, Brazil, 2008.

*Weighted prevalence

BMW = Brazilian Minimum Wage

The outcomes of the unadjusted and adjusted Poisson multilevel models are shown in Table 3 that displays the PR estimates for each level of explanatory variables. After adjustment, HDI, sex, skin color/race, *per capita *income, untreated dental caries, and endodontic treatment need remained significantly associated with dental pain. Age showed a borderline association with dental pain (*p *= 0.064), and education of the father lost statistical significance (*p *= 0.076). HDI remains associated with dental pain prevalence. Adolescents from areas with high human development index showed 26% less dental pain prevalence than those from low IDI areas regardless of individual-level characteristics. An interaction between HDI and *per capita *family income was statistically significant with dental pain prevalence in unadjusted analysis (p < 0.01) but this association disappeared when adjusted for the variables in the multivariable model (p = 0.900).

**Table 3:** Unadjusted and adjusted Poisson multilevel assessment of dental pain period prevalence among adolescents

City level	Unadjusted Prevalence Ratio		Adjusted Prevalence Ratio	
*Human development index*	(95% CI)	p-values	(95% CI)	p-values
Less than 0.48	reference		reference	
0.48 or more	1.62 (1.43-1.84)	< 0.001	0.74 (0.64-0.85)	< 0.001
^_^2-loglikelihood (contextual level)			6963.4	

**Individual level**	**Prevalence** **Ratio**		**Prevalence Ratio**	
	(95% CI)		(95% CI)	p-values

**Demographic characteristics**				
*Sex*				< 0.001
Boys	reference		reference	
Girls	1.62 (1.43-1.84)		1.67 (1.48-1.89)	
*Age (years)*				0.064
12	reference		reference	
15	1.08 (0.93-1.25)		1.14 (0.99-1.30)	
*Skin color/race*				< 0.001
Whites	reference		reference	
Dark-skinned blacks	1.52 (1.24-1.85)		1.52 (1.25-1.85)	
Light-skinned blacks	1.71 (1.46-2.00)		1.69 (1.47-1.95)	
Asian descendants	0.82 (0.47-1.40)		0.83 (0.46-1.48)	
Amerindians	1.42 (078-2.59)		1.46 (0.84-2.53)	
^_^2-loglikelihood (contextual level + demographic characteristics)			6824.1	

**Socioeconomic status**				
*Per capita family income*				0.022
< 1/4 BMW*	reference		reference	
1/4 to 1/2 BMW*	0.88 (0.74-1.05)		0.95 (0.82-1.12)	
≥ 1/2 BMW*	0.45 (0.37-0.55)		0.71 (0.59-0.87)	
Not informed	0.65 (0.55-0.76)		0.89 (0.75-1.05)	
*Father Educational level (years of study)*				0.076
< 8	reference		reference	
8 |-- 11	0.78 (0.65-0.93)		0.92 (0.77-1.10)	
≥ 11	0.46 (0.38-0.56)		0.75 (0.64-0.88)	
Not informed	0.65 (0.55-0.76)		0.93 (0.76-1.13)	
*Mother Educational level (years of study)*				0.038
< 8	reference		Reference	
8 |-- 11	0.75 (0.64-0.87)		0.87 (0.75-1.01)	
≥ 11	0.48 (0.39-0.59)		0.80 (0.67-0.96)	
Not informed	0.73 (0.61-0.88)		0.85 (0.66-1.11)	
*Type of school*				< 0.001
Public	reference		reference	
Private	0.33 (0.22-0.50)		0.50 (0.34-0.73)	
^_^2-loglikelihood (contextual level + demographic + socioeconomic characteristics)			6717.5	

**Dental status**				
*Untreated dental caries*				< 0.001
None	reference		reference	
≥ 1 teeth	2.73 (2.35-3.14)		1.89 (1.63-2.19)	
*Endodontic treatment need*				< 0.001
None	reference		reference	
≥ 1 teeth	3.38 (2.95-3.87)		1.93 (1.70-2.20)	
^_^2-loglikelihood (contextual level + demographic + socioeconomic + dental characteristics)			6464.4	

São Paulo, Brazil, 2008.

*BMW = Brazilian Minimum Wage

## Discussion

This is a school-based study carried out in a Latin-American metropolis, which used a large sample size and international standard procedures for sample selection. Some comments about the study's methodological strengths and limitations are relevant.

The option to choose schools instead households as sample unit was owing to practical reasons. Despite this option, the results may be inferred to the whole population of the same age living in the city, owing to the very high enrolment rates at educational system observed in these age groups: 98.8 and 86.3% for 7-14 years and 15-17 years, respectively, in the state of São Paulo, 2006-2007 [30]. In addition, the survey achieved a high response rate; dental examiners were appraised as highly reliable. The time frame of months adopted in this study was similar to other dental pain population based study [12,31]. Period prevalence, the measurement adopted for the assessment of dental pain, can be conceptualized as the frequency of an existing disease or condition during a defined period of time [25]. Once dental pain is acute in nature, point prevalence, a measure estimate at a point in time, is very low.

Multilevel models were performed to capture both contextual and individual dental pain potential determinants. This assessment followed a conceptual theoretical framework to the selection of covariates. As the outcome was relatively common, statistical analyses used Poisson regression instead of multivariable logistic regression [32].

However, the sample size was neither planned to run multilevel models nor designed to test a specific hypothesis, as was done in a research carried out in Australia to assess neighborhood influences on dental losses [18]. However, we presume that this limitation may have been minimized by the large sample used in this survey. As socioeconomic data were gathered from questionnaires sent to survey participants (parents or guardians), it achieved relatively reduced missing values for specific variables; e.g., 30% for the *per capita *family income. In addition, we cannot assure that adolescent's school and adolescent's households are placed in the same geographical area. However, our results indicate that selection bias is unlikely; dental-pain period prevalence among those adolescents without available information on *per capita *income was nearly equivalent to dental-pain prevalence for the whole studied population (21.3 and 25.6%, respectively). The unavailability of the information about dental care at the geographic area is another limitation of this work.

Dental pain in the last 6 months was a common finding among 12- and 15-year-old adolescents living in São Paulo. Near one-fourths of all the surveyed adolescents reported at least one episode of dental pain. International comparison of dental-pain prevalence studies needs to be carried out carefully owing to the methodological differences among them, such as different age ranges, several options of case definition, difference in the assessment methods adopted, and difference in the time frame. Bailit [33] reported that 5% of 5-12-year-old US children reported dental pain in the previous 3 months. Pau *et al*.[34] reviewed dental pain epidemiological studies and found that 32% of Australian children reported a life-time prevalence; 47.6% of Uganda's 10-14-year-old schoolchildren, using a recall of 12 months, and achieved 30.4% among 11-14--year-old Pakistani who reported at least one episode of dental pain in the last month. Dental-pain prevalence found in our study was lower than that reported by previous research carried out in Northeast Brazil among 14-15-year-old (25.6 and 33.6%, respectively) [14].

Dental pain was associated with contextual and individual socioeconomic status. Contextual Human Development Index was associated with dental pain regardless of individual-level characteristics. Some authors considered dental pain experience as a predictor of dental services utilization and pattern of dental care [35]. The use and availability of dental care should be associated with contextual environment, possibly explaining the findings of our study. However, skin color and sex remained associated with dental pain, regardless of human development level of the region. Previous studies reported a consistent association between the levels of periodontal disease and skin color among adults after adjustment for other socioeconomic variables [36], and a persistent color/race gradient in the prevalence of dental outcomes was observed among adolescents, with the darker the participants' color, the higher the prevalence of unfavorable oral health conditions [37]. The measurement of the aspects of racial discrimination (e.g., chronic psychological stress) or unmeasured factors associated with both skin color and specified outcome, but not related to either discrimination or socioeconomic position, such as culturally shaped patterns of health-related behaviors, may contribute to explain the persistence of skin-color inequalities [38]. In spite of the relationship between gender and dental pain being inconclusive [12], some authors have hypothesized that women are more likely to report pain while males are socialized to suppress signs of pain [39].

## Conclusions

Gender, individual and contextual socioeconomic factors, and dental treatment needs were associated with dental pain in 12- and 15-year-old adolescents. Multilevel analysis revealed that contextual HDI remained associated with dental pain regardless individual-level characteristics. These findings suggest that contextual factors must be considered when dental care are planned and implemented.

## Competing interests

The authors declare that they have no competing interests.

## Authors' contributions

MAP conceived the study, participated in the statistical analysis, and drafted the manuscript. KAP revised the literature and drafted the manuscript. ACF participated in the design of the study. JLFA performed the statistical analysis and drafted the manuscript. All the authors read and approved the final manuscript.

## Pre-publication history

The pre-publication history for this paper can be accessed here:

http://www.biomedcentral.com/1472-6831/10/20/prepub
